# The role of neurology in the development of community healthcare within the Italian national health service. The position of the Italian society of neurology (SIN)

**DOI:** 10.1007/s10072-025-08237-0

**Published:** 2025-06-17

**Authors:** Pietro Ferrara, Vincenzo Andreone, Fabio Bandini, Massimo Del Sette, Marco Longoni, Roberto Marconi, Carlo Alberto Mariani, Francesca Romana Pezzella, Rocco Quatrale, Carla Zanferrari, Maria Luisa Zedde, Alessandro Padovani, on behalf of the Neurology, Healthcare Task Force of the Italian Society of Neurology

**Affiliations:** 1 Center for Public Health Research, University of Milan–Bicocca, 20900 Monza, Italy; 2 Laboratory of Public Health, IRCCS Istituto Auxologico Italiano, 20165 Milan, Italy; 3 Neurology Unit and Strke, Azienda Ospedaliera “Antonio Cardarelli”, Naples, Italy; 4 IRCCS Ospedale Policlinico San Martino, Genova, Italy; 5 Department of Neurology, ASL 3 Genovese, Genoa, Italy; 6 Neurology and Stroke Unit, Bufalini Hospital Cesena, AUSL Romagna, Cesena, Italy; 7 Neurology Unit, Ospedale Di Grosseto, Azienda Usl Toscana Sud Est, Grosseto, Italy; 8 Territorial Assistance Unit “Centro”, Provincial Health-Care Service (ASP) of Palermo, 90141 Palermo, Italy; 9 Neurology and Stroke Unit, ASST Melegnano - Martesana, Vizzolo Predabissi (Milano), Via Pandina 1, Milano, Italy; 10 Stroke Unit, Department of Neuroscience, San Camillo Forlanini Hospital, Rome, Italy; 11 Department of Neurology, Ospedale Dell’Angelo, Venezia, Mestre Italy; 12 Neurology Unit, Stroke Unit, Azienda Unitò Sanitaria Locale-IRCCS Di Reggio Emilia, Vai Amendola 2, 42122 Reggio Emilia, Italy; 13 Department of Clinical and Experimental Sciences, General Neurology Unit, Department of “Continuità Di Cura E Fragilità”, Neurology InstituteUniversity of BresciaASST Spedali Civili of Brescia, Piazza Ospedale 1, 25040 Brescia, Lombardia Italia

**Keywords:** Neurological care, Neurology services, Community healthcare, Continuity of care, Health system organization

## Abstract

Neurological disorders rank within the leading causes of disability and premature death in Europe and Italy, and their prevalence is expected to rise due to the aging population. In Italy, the current approach to neurological care is primarily centered in hospitals. However, an effective community-based neurology care system would ensure that patients receive appropriate care in the right setting, from the right healthcare professional, and at the right time. This “precision” approach would also alleviate the burden on acute neurology facilities. To address the growing demand for neurological care services, experts from the Italian Society of Neurology (SIN) have launched a nationwide initiative aimed at supporting a transformative program in local health and care systems, enabling them to better meet the needs of neurological patients. This document presents a set of consensus recommendations for redesigning neurological care in Italy, with the objective of aligning services with the health requirements of neurological patients and establishing an integrated model for managing neurological disorders.

## Introduction

The direction in which Neurology in Italy is moving in terms of healthcare provision is twofold: on one hand, neurologists are called upon to manage acute neurological diseases primarily in hospital settings; on the other hand, the increasing life expectancy of the population and advancements in both pharmacological and non-pharmacological management of neurological diseases have progressively raised the number of patients requiring chronic care and therapies, making it crucial to shift towards a systemic, appropriate, and sustainable management of chronic conditions.

Chronic diseases affect over 14 million Italians, and according to data from the surveillance of the Italian National Institute of Health, more than half of the population over the age of 65 lives with one or more chronic conditions, with a growing trend as age increases [1].

In general, these conditions represent a significant challenge to the sustainability of healthcare systems and the control of costs, which continue to rise alongside the epidemiological transition, increasing both the number and impact of chronic diseases. In this context, it becomes increasingly essential to optimize and coordinate various levels of care, placing patient needs at the center and ensuring the effective allocation of resources and costs [2].

Specifically, healthcare for chronic neurological diseases poses a growing challenge for social and healthcare systems. According to the most recent estimates from the Global Burden of Disease Study, neurological disorders are the leading cause of ill health and disability and the second leading cause of premature mortality in Europe, with health and social impacts increasing due to the aging population. Among the major chronic neurological diseases are epilepsy, headache, insomnia, neuropathies, myopathies, Parkinson’s disease (and all forms of parkinsonism), Alzheimer’s disease, multiple sclerosis, and, not least, other less common diseases such as encephalitis, meningitis, brain tumors, amyotrophic lateral sclerosis, and genetic neurological diseases [3].

Comprehensive analyses find that the burden of neurological conditions in Italy is not insignificant. Over 7 million people suffer from migraines, while 12 million are affected by sleep disorders, and more than 1,200,000 people live with dementia, 720,000 of whom have Alzheimer’s disease. Over 1 million suffer from disorders related to peripheral neuropathies and myopathies, while 800,000 patients experience the disabling consequences of stroke, a condition that records 180,000 new cases annually. Furthermore, 400,000 people are affected by Parkinson’s disease. It is also estimated that approximately 130,000 people suffer from demyelinating diseases, including multiple sclerosis. Chronic neurological diseases thus represent a complex set of conditions that, due to their nature and duration, require coordinated specialist care. Many services required at various stages of the patient's journey, particularly those of low care intensity, could be managed in non-hospital settings—at the local level or directly at the patient’s home—within the various components of the Chronic Care Model, resulting in better resource allocation and ensuring a high quality of care [4].

Many of these conditions share distinctive characteristics in their care and support processes and models. They are often defined by the chronic nature of health needs, which requires a care approach that ensures continuous and sustained responses. Additionally, the clinical complexity of these conditions, marked by highly variable health demands and often uncertain disease presentations, necessitates specialized expertise, typically provided in dedicated centers or clinics with a focus on accessibility. The care system involved is broad and diverse, encompassing both hospital services and a wide range of local care options.

Moreover, it is essential to recognize that the structure of the neurological care network is not static. External pressures—such as changing epidemiological patterns, local economic constraints, or health policy shifts—can lead to the addition, transformation, or closure of care nodes (e.g., specialized centers or hospital units). Network models should therefore incorporate adaptive governance mechanisms that allow for real-time reconfiguration, ensuring continued access, equity, and efficiency despite evolving care demands and infrastructure.

In summary, the following aspects and their implications are important to consider. First, in ensuring continuity of care, it is necessary to balance the person’s need for a stable relationship with professionals with the potential need for specialized services, which may not always be provided by the usual professional or reference structure. Moreover, the affected person, during the course of the disease, may go through different stages of the somatic condition with profoundly different clinical and care needs: hence, the need to design services as a continuum, carefully planning and monitoring the patient’s transition from one care setting to another, ensuring continuity of care and providing service models that are diversified and consistent with the different stages of the disease. Finally, the progressive emergence of chronic conditions, or phases of chronicity, characterized by high clinical complexity, requires rethinking how to make available chronic care and local and home care settings with services and expertise traditionally associated with acute care, which are inadequate in meeting new and emerging needs.

## Clinical-care networks

From this perspective, clinical networks have become an essential approach for the functioning of healthcare systems [5]. Over time, the term “network” has evolved into a broad concept that encompasses various models and experiences. In general, the concept of a healthcare network, as implemented in the Italian experiences, highlights the need to ensure, in certain areas (needs, diseases, specialties, etc.), levels of coordination that general programming mechanisms at different levels are unable to achieve.

Networks integrate and influence the institutional structure of the National Healthcare Service (NHS), represented by health agencies in their various configurations. The Ministerial Decree No. 70/2015 on the standards related to hospital care outlined the concept of network, which aims to achieve several objectives, including care effectiveness, resource efficiency, and equity in diagnostic and therapeutic opportunities through better coordination among all subjects and nodes involved. Moreover, networks leverage potential synergies, addressing many of the previously identified contradictions [6].

In healthcare, a network thus represents a project that applies to two closely interconnected dimensions: the structure of the service provision and the patient pathways. Designing the functioning of a network means, on one hand, defining the characteristics that the contexts tasked with providing and delivering services (the network nodes) must have and, on the other hand, determining how, and in what sequences, patients move through the system and receive services (the clinical-care pathways). To ensure effectiveness and exploit synergies, the role of each network node, in terms of services provided and conditions treated, must be the result of a conscious choice and not simply the outcome of spontaneous dynamics. Network configurations can vary, ranging from “flat” models, in which each node replicates the functions of others, maximizing proximity for patients, to highly specialized configurations, where differences and distances increase, and each node fulfills specific functions.

These two extreme models serve to guide choices within real contexts, where the network of services is influenced both by the diversity of disciplines involved in the diagnostic and treatment processes and by the interdependencies between clinical and diagnostic services, which are often crucial in the patient pathways. Beyond theoretical assumptions, all networks aim to develop some form of specialization, both horizontally (by disease areas) and vertically, in functions related to a particular health issue.

Specialization, necessary to ensure adequate expertise and access to technological resources, almost inevitably leads to a hierarchy (hub and spoke), though this need not apply to all operational units. It is, in fact, possible for different roles to coexist within the same unit in different networks, or for specialization to be accompanied by shared specialized expertise among units (itinerant teams).

In addition to the degree of specialization and the spatial distribution of nodes, the internal composition and connectivity of the network play a critical role in determining its efficiency. Beyond assortative configurations, it is worth considering the potential advantages of a ‘disassortative architecture’ within the neurological care network. In such models, hubs connect with a diverse range of nodes from different specialties, facilitating cross-disciplinary interaction. The inclusion of nodes representing non-neurological expertise—such as geriatrics, psychiatry, internal medicine, and rheumatology—could significantly enhance care for patients with multimorbidity, frailty, or overlapping syndromes. This integration may promote more holistic care delivery, streamline referrals, and reduce redundancy by enabling more appropriate first-contact responses within the network. Embracing structural heterogeneity in this way may ultimately improve both the robustness and adaptability of the neurological care system.

The design of the roles played by the various network nodes is accompanied by the definition of the pathways that patients follow to obtain responses to their specific needs. Identifying these pathways, from diagnosis to treatment to rehabilitation, and from socio-health needs to possible rehabilitation, better defines the functions of each node and highlights the connections between the network and the healthcare system. Patient pathways must be based on guidelines, protocols, and, in general, a shared approach and care strategy by the relevant scientific community, which are the goals that the clinical-care pathway seeks to promote. From this perspective, clinical-care pathways are one of the most important tools in clinical governance, aligning, at the professional level, the sequence of necessary interventions and coordinating the contributions of the various disciplines in the different stages of the disease. Additionally, comparing actual (real and current) pathways with ideal ones (the design) provides a tool to stimulate and guide change.

Networks are not limited to the design of nodes and the pathways between them, but they must also create the conditions for functioning and evolving. A fundamental element in this regard is network governance, which determines how the network is managed and how it maintains balance dynamically. Various configurations are possible, but it is rare for a network to function without some fundamental elements, which, while potentially compensating for each other, must be present to some degree. Consensus and cohesion within the professional community are key factors; however, it is worth noting that coordination and cooperation are difficult to impose on overly divided professional communities.

As part of this governance structure, the inclusion of all relevant stakeholders—including public providers such as IRCCS and Academies as well as private healthcare providers—requires careful consideration. Their integration into the clinical care network represents both a strategic opportunity and a necessary consideration for system efficiency. Private institutions can play a complementary role, particularly in regions with high demand or limited public infrastructure, by helping to reduce waiting times, expand access, and contribute to quality benchmarking. However, their participation must occur within a regulated framework to ensure alignment with the public health sector’s objectives of equity, appropriateness, and continuity of care. This requires shared clinical protocols, standardized referral pathways, and transparent reporting mechanisms. In fact, a hybrid model—in which private providers contribute to predefined segments of the network, such as elective outpatient care or non-urgent diagnostics—may enhance capacity without fragmenting patient pathways. Strategic governance will be essential to ensure that private sector engagement supports, rather than undermines, the coherence and public value of the integrated neurological care system.

Over the past four years, the COVID-19 pandemic has accelerated the development and availability of proximity healthcare services, improving patient access to healthcare services. This trend is considered a priority by policymakers, starting with the national government, and is one of the pillars of the National Recovery and Resilience Plan (PNRR), which is also focused on strategies to better serve high need patients in a more robust and sustainable NHS with a particular focus on strengthening community healthcare, by virtue of the Next Generation EU plan of European Union, a temporary instrument to boost the recovery in the post-COVID-19 Europe.

Specifically, the “Health Mission” of the PNRR focuses on proximity networks, healthcare facilities, and telemedicine for community healthcare, with the general aim of aligning services with the needs of communities and patients, improving home care, overcoming the fragmentation and lack of uniformity in healthcare services offered across the territory, developing telemedicine with advanced solutions to support territorial and home healthcare.

The experiences already established in the country offer a wide range of tools to support networks for neurological diseases within hospital and territorial neurology structures. Currently, networks for chronic neurological diseases are structured through a set of disease-specific networks revolving around hospital Neurology Units (Complex or Departmental Structures). Indeed, within the healthcare system, there is a well-established wealth of expertise and structures dedicated especially to the diagnosis and treatment of dementias, Alzheimer’s disease, Parkinson’s disease and parkinsonisms, headaches, and epilepsy. These networks, which are deeply rooted within hospital Neurology Units (for example, networks of clinics for Multiple Sclerosis and Centers for Cognitive Disorders and Dementias), require strengthened collaboration with other professional communities and greater integration with the network of territorial services and offerings.

While the implementation of standard procedures and a systematized network can promote equity and consistency of care, it is important to acknowledge a potential drawback: the risk of weakening the continuity of the physician–patient relationship. In distributed care models, patients may encounter different neurologists across various nodes of the network, which could lead to a more fragmented and less personalized experience. This has implications not only for clinical continuity but also for the establishment of an empathic, trusting relationship—an essential element of neurological care, particularly in chronic and progressive conditions. To mitigate this, the model should include mechanisms to preserve relational continuity, such as structured follow-up by the same clinical team, the use of shared electronic health records, and regular interdisciplinary case reviews. These strategies can help ensure that patients experience care that is not only clinically coherent but also personally meaningful.

## Care settings between hospital and community

The organization of the neurological network should be structured to ensure close interaction and the sharing of organized pathways between hospital and community settings.

This approach should meet several needs, including:

- Ensuring comprehensive care for patients affected by major neurological diseases;
- Ensuring continuity of care, adapting the provision of services to the epidemiology and natural history of neurological diseases;
- Reducing unnecessary hospital admissions and avoiding unjustified hospitalizations;
- Promoting a shared training pathway for all network nodes, encouraging active participation and maintaining consistent and uniform quality standards;
- Defining differentiated levels of clinical and care complexity, with clear access criteria for first and second-level outpatient clinics;
- Shifting non-urgent neurological consultations to community settings, thereby decongesting emergency departments;
- Launching a working group on prescription appropriateness, involving key system stakeholders;
- Ensuring better resource allocation and avoiding waste.

The community and hospital settings should work together to promote operational protocols and clinical-care pathways that support the development of neurological specialist community teams, closely integrated with second-level centers. Additionally, these pathways should encourage the involvement of other healthcare professionals in managing dedicated care for specific diseases.

The foundations of this organization derive from the identification of certain critical issues: i) inadequacy of the service provision relative to the epidemiology of individual diseases, with long waiting times; ii) geographical disparities in access to dedicated centers, with patients foregoing care due to the distance from their residence; iii) lack of a clear interface between community neurology and dedicated hospital centers; iv) poor differentiation between first- and second-level activities within the same center.

A network organization for neurological (chronic) diseases is proposed based on common principles as listed in Table 1 dealing with neurological formation, integration between hospital and community settings, services’ provision, access equity and patients’ involvement.

**Table 1:** List of proposed principles for the organization of a neurological chronic disease network

a. Formation of a homogeneous group of neurologists dedicated to specific diseases, with differentiated activities between first and second levels based on complexity
b. Overcoming the physical division between hospital and community, allowing contracted specialists to integrate into the second hospital level
c. Adoption of shared clinical-care pathways and protocols d. Uniform and targeted professional growth for physicians and other dedicated figures
e. Adequate service provision aligned with the epidemiology of diseases, ensuring the availability of services and thus waiting times that are compatible with clinical needs
f. Overcoming geographical disparities with greater equity in access through the creation of first-level outpatient clinics in peripheral areas
g. Involvement of patients and caregivers in gathering needs

For all the above categories, the network pathway can be structured into two levels based on clinical complexity and stability, with access determined by predefined criteria related to disease type and treatment needs—namely, first-level outpatient clinics (primarily community-based) and second-level outpatient clinics (primarily hospital-based).

Defining the pathways would not only make the system more efficient, improving the quality of care and facilitating the patient experience, but also help estimate the increase in demand and enable targeted and timely intervention by adjusting service provision based on identified needs. For example, services could be scheduled based on epidemiological data and aligned with referral protocols that ensure appropriateness, thus addressing growing demand while avoiding unnecessary access.

Within the network context, innovative pathways for disease groups, sharing common socio-assistive needs, or disease-specific pathways, including neurophysiological and neurosonological diagnostic pathways (first and second levels), could find justification.

For instance, pathways for disease groups may include those dedicated to rare diseases. While these conditions have limited impact on overall healthcare system efficiency or costs due to their low prevalence, they nonetheless provide a useful model for designing coordinated, equitable, and high-quality care pathways. They underscore the need for structured referral criteria, specialized centers, and the integration of second-level outpatient clinics with hospital or day-hospital services. Their complexity justifies a network approach that, while not system-defining, serves as a benchmark for managing diagnostic uncertainty and clinical fragility.

Similarly, when considering disease-specific pathways, one can look at the example of stroke and post-stroke care pathways, integrated with cerebrovascular clinics in the hospital setting dedicated to post-discharge care from Stroke Units. In these clinics, the need and timing of a follow-up visit are defined at discharge. There is also the possibility of access for selected patients referred by other neurologists, who were not discharged from the Stroke Unit, following case discussion. Patients who do not need dedicated second-level follow-up, either initially or after the follow-up visit (e.g., at three months), are then referred to the district General Neurology clinic (first level).

In general, access methods should be defined between the levels of care described above, in line with the guidelines outlined here.

Access methods to the first level would be managed through the COT (‘Centrale Operativa Territoriale’) which coordinates with all relevant services and the emergency system. This coordination is supported by information systems and telemedicine tools and utilizes the CUP (‘Centro Unico Prenotazioni’, Unified Booking System) for appointments with hospital or community neurologists.

Similarly, access to second-level care would also be facilitated by COT through CUP booking with a dedicated agenda, utilizing specific keywords based on referrals from General Practitioners (GPs), hospital and community neurologists, or other specialists in accordance with the relevant guidelines. Additionally, first-level neurologists could arrange CUP bookings or teleconsultations with second-level specialists, accompanied by essential documentation to define the appropriate care pathway.

Coordination between first and second levels would include meetings where physicians and other specialists or operators particularly involved in the pathway meet to discuss complex clinical cases, implement diagnostic and therapeutic innovations, continuously improve the pathway with a view to building clinical-care pathways in accordance with specific guidelines, evaluate specific training opportunities, and monitor indicators and assess changes to the pathway.

The first and second levels for each disease are defined, where available, based on the indications of the relevant scientific societies, also considering general complexity (as well as indicators of frailty) and specialist complexity. Where possible, it is also crucial to define healthcare organizational models that consider the voice of patients, involving associations and using tools such as Patient-Reported Outcome Measures (PROMs) and Patient-Reported Experience Measures (PREMs) as quality indicators. These tools allow for assessing the effectiveness of services delivered in various settings, ensuring that care is truly centered on patients'needs and experiences, thus improving overall care.

The proposed organizational steps should be followed by the development of advanced analytical tools for evaluating and managing care, with a focus on flexibility and adaptability to the specific local characteristics. These tools should allow for accurate monitoring of healthcare performance and the quality of care provided, considering the specific demographic, epidemiological, and socioeconomic characteristics of each area.

## The models and standards of ministerial decree 77 of 2002: structuring a hospital–community network for neurological patients

The issuance of Ministerial Decree 77 of 2022 defined the models and standards for the development of community healthcare in the National Health Service [7]. The Decree provided concrete recommendations that allowed the project to proceed more effectively in formulating innovative and, above all, necessary proposals to adequately address the challenges posed by the current healthcare needs of the population. One of the central elements of this reform in territorial healthcare is the District, a territorial organizational structure of the local health authorities into which the Italian NHS is divided. Each District is designed to serve a population of 100,000 inhabitants. It serves as the reference point and coordination level for the delivery of healthcare services within community healthcare facilities. Among the structures and units encompassed within the District, new social-healthcare facilities have been introduced, adding to the existing territorial structures (i.e., Community Outpatient Settings) and becoming an integral part of the NHS: the Community Health Centers (‘Casa di Comunità’, CdC) and the Community Hospitals (‘Ospedale di Comunità’, OdC). The first is defined as a physical, easily identifiable location, primarily intended to manage both chronic conditions and complex health and social needs, where citizens can access healthcare and social-healthcare services, and it represents the organizational model for proximity care for the reference population. In it, all professionals work in an integrated and multidisciplinary manner to design and deliver healthcare and social integration interventions in a citizen-centered proximity framework. The CdCs are divided into two levels of complexity and service provision, following a Hub-and-Spoke model. The Hub, planned for every 50,000 inhabitants (thus two per district), serves as the central point of coordination. The Spoke functions as a more peripheral and decentralized structure, closely linked to the Hub CdC Community Health Center Hub, with more limited and focused functions [8].

The OdC is a healthcare facility within the community healthcare network, serving as an intermediate step between home and hospital care, planned for every 50,000–100,000 inhabitants. It is aimed at patients who, following a minor acute episode or a flare-up of chronic conditions, require low-intensity clinical healthcare interventions that could potentially be delivered at home but need continuous nursing care and supervision, including overnight, which cannot be provided at home or where the home setting is inadequate. OdCs will be managed by nursing staff, while medical care is provided by GP, with access to consultations from outpatient specialists [7].

The new organizational frameworks introduced by CdC and OdC are, in some contexts, integrated with pre-existing territorial outpatient facilities, which, in accordance with regional differences within the Italian NHS, were already in place.

**Fig. 1:**
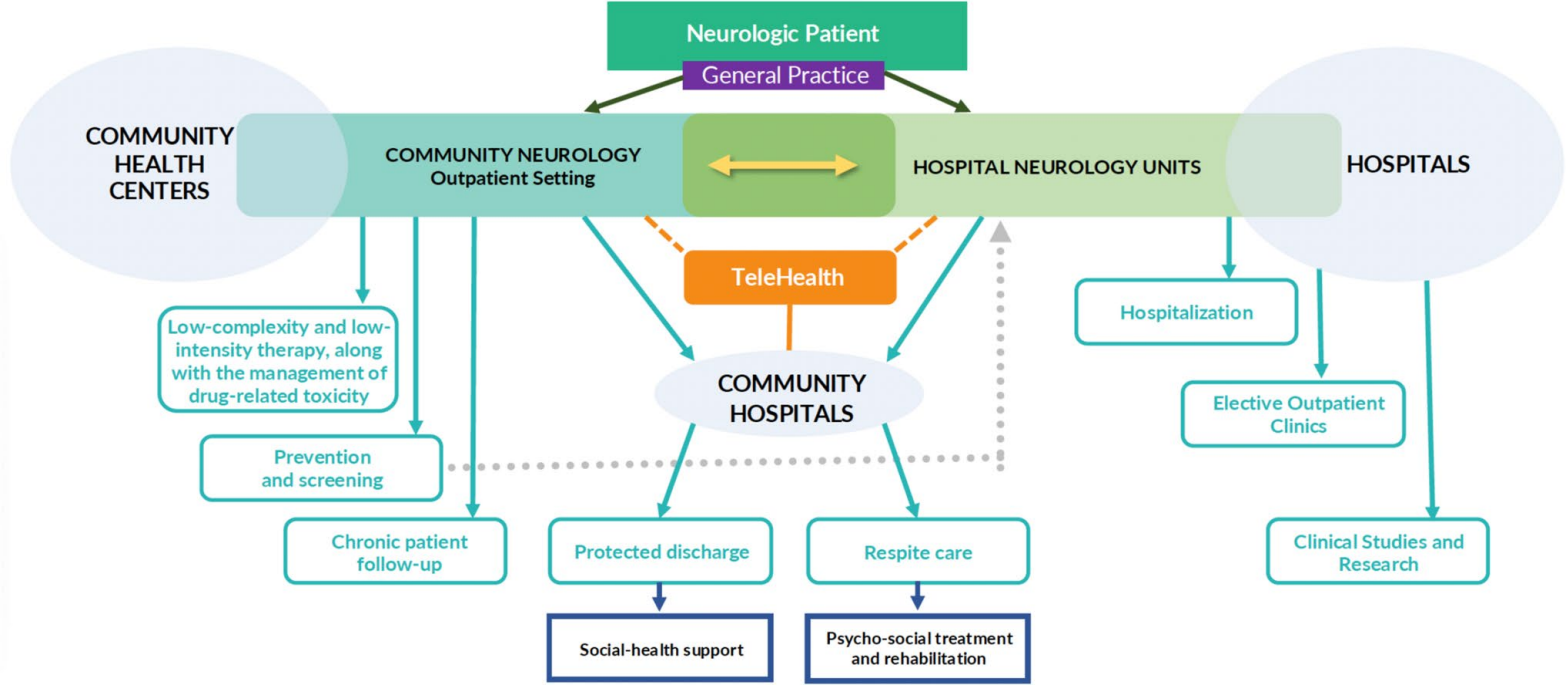
The neurologic patient within the hospital-community network

In the new service architecture, central is the contribution that CdCs and OdCs, and the intensification of home delivery models can offer, together with forms of telemedicine, digital health, and mHealth. The network organization enables the management of interdependencies not only between the contributions that various actors can offer patients at different stages of illness but also those related to the integration of healthcare and non-healthcare services for the person and support for their family, aiming for a true community-based neurology. This occurs in close interaction and with a shared organization of care pathways, between hospital and community settings. Outpatient neurologist specialists can be functionally integrated into the Neurology Units. Figure 1 illustrates how, with the new healthcare service architecture, patients with neurological symptoms or diagnoses can have their needs addressed within the hospital-community network.

The redesign of neurological services aims to optimize patient management, reduce fragmentation, and improve care efficiency. Among the main benefits is the limitation of access to pathways with a risk of inappropriate instrumental prescriptions and the reduction of repeated procedures, resulting in better resource utilization. These models also allow outcomes to be measured at the network level, rather than focusing solely on individual clinics, offering a broader view of the system’s overall effectiveness. Furthermore, the possibility of encountering the same professionals during both scheduled outpatient visits and emergencies improves continuity of care, further limiting over-prescription. Ongoing training of staff within the network ensures a coordinated and up-to-date approach.

However, there are also some challenges related to the large-scale application of these models, particularly in areas characterized by high local and interregional mobility within the healthcare system, which may require specific adaptations. Another potential drawback may result from the differing coordination among health care structures—such as hospitals, departments, and community services—which can complicate efforts toward effective integration.

Regarding future developments, the introduction of teleconsultations for certain categories of patients could facilitate access to care, while direct contact with GPs could improve the timeliness of interventions. The development of neurology urgent care clinics (Fast Track Day Service) with priority access within 72 h could ultimately reduce inappropriate emergency department visits, optimizing care for neurological emergencies. To evaluate the feasibility of the proposed advanced and innovative solutions, the organizational model should be supported by an assessment framework capable of measuring its technical, allocative, and social value through meaningful indicators (see Table 2).

**Table 2:** List of useful indicators to assess technical, allocative, and social value of the hospital-community network organization

a. Waiting times for access to a first neurological visit
b. Volume and modality of communication between first- and second-level clinics, including the number of contact forms, email exchanges, and phone consultations
c. Appropriateness in referral prioritization, measured by the correct attribution of urgency classes (U, B, D, and P) based on clinical information shared across levels
d. Number of shared training activities involving GPs, specialists, family and community nurses, and other healthcare professionals
e. Frequency of multidisciplinary audit meetings between hospitals and Community Health Centers
f. Formalization of clinical-care pathways, including measurable indicators and standardized referral criteria
g. Proportion of patients accessing first-level clinics with an appropriate CUP referral, based on predefined indications
h. Patient satisfaction and perceived quality of care, as assessed through structured feedback tools

## Proposal for a new organizational model

Italian regional healthcare services exhibit considerable heterogeneity in their organization, reflecting the socioeconomic, demographic, and cultural differences across various geographic areas. Regions have different organizational models, influenced by local and national policies, available resources, and the specific needs of the population. In some regions, there is a greater emphasis on accessibility to primary care services, while in others, a more centralized model with larger hospital structures may prevail. Variations may also concern the types of community and home care services, which may be focused on managing chronic conditions and non-self-sufficiency. The diversity in regional healthcare services reflects the need to adapt the various guidelines of this document to local specificities to ensure appropriate, integrated, and patient-centered care in every regional and local context.

The implementation of the guidelines contained herein must, however, consider two important factors: the intensity of care and the complexity of services, as well as the regional organizational specificities. Based on these premises, it is possible to define the activities and actions that are prime candidates for decentralization, identifying those services that can be provided in community facilities, ensuring both safety of care and sustainable resource management. This aims to ensure the provision of proximity healthcare services, where community-based healthcare providers work in synergy with patients to develop personalized care plans that integrate chronic disease management with preventive interventions. Additionally, community healthcare promotes continuity of care by fostering collaboration between GPs, specialists, nurses, and other healthcare professionals.

While regional and subregional care networks are essential for ensuring proximity and continuity of care, it is equally important to formally recognize and structure long-distance interregional connections within the national healthcare framework. Certain neurological conditions—such as central nervous system vasculitis, pediatric neurological disorders, or rare diseases requiring highly specialized diagnostics—often necessitate referral to centers of excellence located outside the patient’s region of residence, particularly in the smaller regions of the country. These referrals should be guided by clear national protocols and equity-based access criteria, ensuring that all patients, regardless of geographic location, might benefit from timely access to advanced diagnostic and therapeutic services. This approach should promote uniform standards of care across the country and helps reduce disparities stemming from regional fragmentation in specialist expertise.

Regarding specialist personnel, considering all the various neurological conditions that make up the Neurology ecosystem, and recognizing that in many cases, symptoms and signs related to the central and peripheral nervous systems do not necessarily require elective neurological care, it is deemed preferable in the current context to establish neurological teams primarily composed of specialist neurologists, through shared governance mechanisms across different care settings.

Based on current demographic trends and the number of projected complex/frail/disabled cases [9], the total number of decentralizable neurological patients followed at community settings is estimated to be around 20,000 people per standard District, many of whom will be eligible for management in CdCs, according to the organizational models and available resources.

The criteria for the decentralization of neurological patients to CdCs must be defined in collaboration with the hospitals and GPs from the districts, to ensure proper patient management and optimal continuity of care.

The Ministerial Decree 77 of 2022, based on common population health models, proposes a six-level population stratification model, which involves dividing the population into homogeneous groups based on specific characteristics (e.g., age or the presence of chronic conditions) to identify the homogeneous healthcare needs of each group [7]. The objective of stratification is also to provide a basis that could be exploited to design targeted and personalized healthcare interventions that consider the specific needs of the various population groups. Based on this outline, Table 3 outlines criteria for stratifying the neurological population, which help in identifying the patients who can be more properly managed in community-based facilities.

**Table 3:** Criteria for stratifying the neurological population with the ideally most appropriate care setting based on socio-health needs profiles and risk

Level	Description *	Population proportion *	Setting for neurological care
I Healthy population	Individuals without neurological conditions or care needs	45%	These are patients who primarily consult their GPs in cases of new and specific symptoms. They are also the target of primary prevention campaigns and health promotion initiatives conducted by primary care services
II Person with minimal or short-term clinical and care complexity	These are users who sporadically utilize services, limited to a single clinical episode	30–35%	Neurological outpatient settings or GPs for primary/secondary prevention and screening programs
III Person with moderate clinical and care complexity	It includes patients with early-stage chronic mono-pathology (e.g., migraine, epilepsy, Parkinson disease, chronic severe insomnia, rare neurological disorders, ALS) requiring self-care support, frequent monitoring. As far as social and health demand is concerned, patients suffering from neurological conditions in the early stages may fall into this group and therefore need services of a predominantly diagnostic nature and accompaniment with support to the caregiver (19% patients/users)	15–20%	These are patients who, due to diagnostic assessment and therapeutic planning, require access to specialized and excellence centers (disease-specific centers, hospital outpatient clinics), with referral to community settings (i.e., CdCs) for low-intensity cases and follow-up care
IV Person with medium–high clinical and care complexity, with or without social frailty	It includes polypathological patients, with already complicated chronic diseases or with several concomitant morbid conditions (e.g. Parkinson disease, Alzheimer Disease, Cerebrovascular Disease) that require frequent intervention by the outpatient specialist for follow-up and stabilization of the disease. The management of these patients requires continuous connection between several professionals at the specialist and territorial level	10–15%	Community settings for follow-up, with access to hospital specialist outpatient clinics for the management of acute conditions and relapses
V Person with high clinical and care complexity, potentially with social frailty	It includes various morbid conditions of such complexity (e.g. stroke, encephalitis, multiple sclerosis) that they require hospital care (in emergency-urgency or in high-intensity care or highly specialized wards), a long rehabilitation phase and territorial follow-up in cases where the acute phase is resolved	1,5–2%	Hospital (Neurology Unit) according to severity and complexity; Community Hospital for the post-acute phase
VI End-of-life or terminal population	It includes people in the terminal or advanced stage, for whom no further curative options are available, who require predominantly palliative healthcare needs	1–2%	They are the recipients of palliative care, provided in home-based settings or dedicated facilities (such as nursing homes and hospices)

*The sum of the percentages does not add up to 100 because the discriminatory nature of the represented categories also depends on the type of characterization criteria. The values are only intended to guide the relationships between levels

Abbreviations: CdC, Community Health Centers (‘Casa di Comunità’, in Italian); GPs, general practitioners

**Fig. 2:**
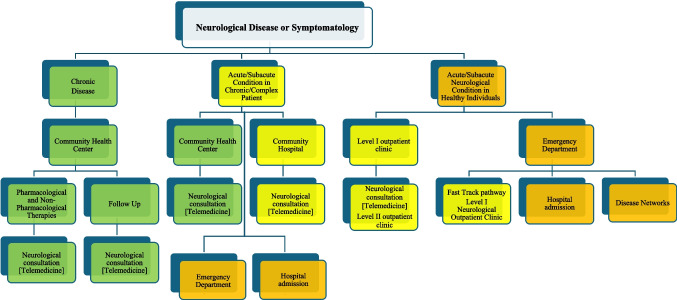
Differentiated neurological pathways based on care needs

The organization follows the model described in Fig. 2, which outlines differentiated pathways based on care needs, the stratification of the patient’s condition, and urgency/emergency situations.

## Neurological services in the community

The activities and actions identified for decentralization in community healthcare facilities may include neurological consultation, cognitive assessment, diagnostic exams, drug administration, and rehabilitation care. In general, the activity of the Neurology Specialist would be dependent on the setting and level of complexity of patients as follows.

### Neurological specialist consultation

#### Community outpatient setting

At this level of care, patients with de novo neurological symptoms/signs of low intensity or chronic conditions without severe disability, high fragility, or complexity can access services. The services that can be provided at the Community Outpatient Setting are:

- **Neurological consultation (first visit):** Excluding urgent visits or those requiring high-intensity care, a first neurological consultation for diagnostic assessment and therapeutic management can be performed at Specialist Community Outpatient Clinics or Divisional Outpatient Clinics at the Hospital. Neurological evaluation is carried out using standardized tools. The organization provides for the creation of ad hoc diagnostic-care pathways for referring more complex cases to second- or third-level clinics, day-service centers, or day hospitals, as well as for patients with moderate to severe complexity or subacute conditions.
- **Follow-up and control neurological consultation (including home visits):** This service is mainly provided in the community for neurological cases where the diagnostic process is concluded. In community outpatient clinics, follow-up activities via telemedicine (e.g., teleconsultation and monitoring through digital tools or self-assessment PROMs) are planned for cases of low complexity, fragility, comorbidity, and intensity. Neurological evaluation requires the use of standardized tools, even in the case of teleconsultation.
- **Cognitive and behavioral assessments:** Cognitive-behavioral evaluation can be carried out in the community, typically with appropriately trained personnel (psychologist with cognitive expertise) using standardized evaluation tools, including digital tools. Cognitive-behavioral assessment is necessary for a possible multidimensional evaluation at Centers for Cognitive Disorders and Dementias or for referral to a Cognitive-Behavioral Neurology Unit in cases unrelated to dementia, or to Mental Health Centers for psychiatric disorders.

#### Community health center setting

CdCs are the appropriate care setting for patients with prevalent or new chronic neurological symptoms/signs, accompanied by moderate to severe disability and fragility, and general complexity. Services provided at CdCs include:

- **Neurological consultation (first visit):** Excluding urgent visits or those requiring high-intensity care, a first neurological consultation for diagnostic assessment and therapeutic management can be performed at Community Health Centers, both Hub and Spoke, if the emerging neurological condition occurs in individuals already followed at the Center. Neurological evaluation is carried out using standardized tools. The organization provides for the use of telehealth (on-line visit, teleconsulting, etc.) and the creation of ad hoc diagnostic-care pathways for referring more complex diagnostic cases to the elective centers (second and third levels, or day-service or day-hospital centers).
- **Follow-up and control neurological consultation (including home visits):** This service is mainly provided in Hub CdCs, especially for complex cases where the diagnostic process is concluded. In the CdCs, follow-up activities via telemedicine (e.g., teleconsultation and monitoring through digital tools or self-assessment PROMs) are planned for cases of high complexity, fragility, and comorbidity, with low to moderate neurological intensity. Multidisciplinary teams, including specialists in geriatrics, oncology, cardiology, pulmonology, and other health professionals (e.g., nutritionists, physiotherapists, nurses, speech therapists, psychologists), are important. Neurological evaluation requires the use of standardized tools, even in the case of teleconsultation.
- **Cognitive-behavioral assessment:** Cognitive-behavioral evaluation can be carried out in the community by appropriately trained personnel (neurologist with cognitive expertise, psychologist with neuropsychological expertise).
- **Outpatient clinics** dedicated to managing high-incidence or high-prevalence diseases, regulated within chronic care pathways with dedicated agendas, managed by community or hospital neurologists with certified expertise. These clinics must ensure the prescription of all medications related to the reference disease (including therapeutic plans).

### Neurological diagnostic procedures

Based on the technology and equipment available in the Community Outpatient Setting or CdCs (particularly in the Hub CdCs), it is possible to activate diagnostic pathways for neurological diseases, particularly chronic diseases, and for low-intensity diagnostic examinations (e.g., basic neurophysiological studies and neurosonological TSA exams). These pathways must be activated in coordination with specialized hospital centers to define which exams can be decentralized to individual facilities and to refer patients for follow-up diagnostic (with second-level exams) and therapeutic care. For exams that require it, the presence of a technician in the CdC team is essential.

### Treatments

In community facilities, Community Outpatient Setting or CdCs, equipped with appropriate environments for drug administration, specialized nursing staff, and emergency personnel, it is possible to provide low-risk and low-toxicity pharmacological treatments, as well as the delivery of medications for home use. Treatments can be administered under the supervision of a general practitioner in collaboration with the specialist, also via telemedicine. These treatments are intended for stable patients who are already undergoing treatment, following pathways defined with specialized/hospital centers. Decentralizable treatments include subcutaneous and infusion therapies of low to moderate intensity, as well as monoclonal antibody therapies.

### Specialist neurological rehabilitation care

The rehabilitation of patients within the community can take place across various territorial settings, organized according to the intensity of care required.

#### Community outpatient setting

In Community Outpatient Settings, care can be provided to low-complexity cases, such as individuals with motor or cognitive impairments or disabilities requiring only one type of rehabilitative service. These services are delivered either directly by a neurologist with certified rehabilitation expertise or by rehabilitation professionals, including home-based care.

#### Community health centers and community hospitals

The new structures introduced by the 2022 regulation, namely CdCs and OdCs, also ensure the management of high-complexity cases. These include fragile individuals with significant, often multiple, motor or cognitive impairments or disabilities, requiring long-term comprehensive care involving multiple therapeutic programs provided by rehabilitation professionals. Rehabilitative activities for such cases may be delivered as complex and coordinated outpatient services.

## Network communication and connection

Among the different levels of the network, it is necessary to enable tools for the continuous communication and connection, guaranteeing the understanding and integration between community-based and hospital services. IT, digital health and telehealth tools aim to share and archive clinical information and health data among the different levels of the network, as well as to support referrals and improve the quality of care: the patient pathway must be integrated with a digital pathway. The functioning of the new model of care will therefore depend not only on the implementation of the interventions proposed in the new architecture of the network, but also on the technological and digital investments for the construction of a telehealth framework that guarantees communication and information sharing.

Other examples of connection and coordination between care levels concern the use of effective clinical governance tools, including clinical and referral pathways and protocols between social services of the municipalities.

## Relations with the general practitioner and patients’ involvement

GPs serve as the initial point of contact for patients seeking healthcare assistance and play a pivotal role within the Italian NHS. However, primary care and general practice currently face several structural challenges, including limited integration and coordination with other services, workforce shortages and aging, inadequate specialist referral systems—such as those based on the Homogeneous Waiting Groups criteria—and gaps in training and resources. Despite these obstacles, GPs remain crucial in the neurological care network as the first contact and key coordinators of access to specialist services. Their ability to recognize neurological disease by signs and symptoms, apply standardized referral criteria, and guide patients through the care continuum is essential for ensuring appropriateness and efficiency. Without such a structured approach, simply increasing outpatient availability would risk exacerbating demand and further straining specialist services. Therefore, continuous education and training of GPs on neurological conditions, the adoption of shared referral protocols, and the use of digital decision-support tools should be integral components of the care model, aiming to optimize patient flow and reducing inappropriate access. Strengthening collaboration among GPs, community and hospital neurologists, and other healthcare professionals will also foster mutual learning and improve alignment in clinical practice, ultimately benefiting patient outcomes.

In parallel with professional training and system reorganization, public education campaigns are essential to support the effective functioning of the neurological care network. These initiatives should inform patients and caregivers about the correct access pathways, the rationale behind referral protocols, and the importance of coordinated care over individual provider preference. By discouraging inappropriate self-referral and the insistence on seeing specific clinicians outside established pathways, such efforts can help reduce inefficiencies, improve equity, and preserve the intended logic of the networked care model.

Finally, involving patients and listening to their voices is essential in structuring organizational models and healthcare pathways. The direct experiences of patients, gathered through patient associations and the direct analysis of data (such as PROMs and PREMs), provide a unique and, nowadays, indispensable perspective on the real needs and challenges of the healthcare system. Integrating these opinions into decision-making processes not only improves quality and optimizes service effectiveness but also ensures that care models are truly centered on individual needs. The contribution of patients enables the development of more personalized and inclusive care pathways, tailored to address the specific needs of everyone, while promoting a more equitable and sustainable healthcare system.

## Conclusion

Addressing the burden of neurological disorders is a core pillar of ensuring healthcare and social sustainability. This calls for the development of a new healthcare strategy that aligns services with the specific needs of neurological patients. In the Italian context, Ministerial Decree 77 of 2022 offers a critical opportunity to modernize the country’s community healthcare system, but its success depends on its concrete implementation, the establishment of a performance evaluation system within primary care settings, and the ability to address persistent challenges faced by GPs, including professional isolation, communication gaps, workforce shortages, and resistance to organizational change. To be effective, solutions must prioritize systemic integration, digital innovation, workforce development, and sustained investment—particularly in strengthening relationship and referral processes between GPs, community facilities, hospitals, and specialists.

The innovations outlined in this document are intended to redesign specialist care by implementing an integrated care neurological care model that ensures high-quality standards while adapting to patients’ clinical and social complexity. The proposed organizational framework for managing persons with neurological disorders recognizes the complexity involved in neurological care and holds significant implications for healthcare practices by: i) achieving an integrated care model which aligns services to the health and social needs of neurological patients and their caregivers; ii) facilitating patient’s access by optimizing contacts with the healthcare providers, and thus avoiding improper resources consumption and reducing avoidable costs associated to neurology; developing advanced tele- and e-health solutions to support intermediate (near-the-patient) and home (direct-to-patient) care. Overall, the project emphasizes its public value by offering strategic coordination to inform policymakers and stakeholders. Its primary goals are to enhance patient access to high-quality care and optimize resource allocation within the Italian neurological care network and NHS.

## Data Availability

Not applicable.
